# A randomized controlled trial of a postdischarge nursing intervention for patients with decompensated cirrhosis

**DOI:** 10.1097/HC9.0000000000000418

**Published:** 2024-04-26

**Authors:** Malene Barfod O’Connell, Anne Brødsgaard, Maria Matthè, Lise Hobolth, Laus Wullum, Flemming Bendtsen, Nina Kimer

**Affiliations:** 1 Gastro Unit, Medical Division, Copenhagen University Hospital Amager-Hvidovre, Hvidovre, Denmark; 2 Department of Paediatrics and Adolescent Medicine & Gynaecology and Obstetrics, Copenhagen University Hospital Amager-Hvidovre, Copenhagen, Denmark; 3 Nursing and Health Care, Institute of Public Health, Aarhus University, Aarhus, Denmark; 4 Department of People and Technology, Roskilde University, Roskilde, Denmark; 5 Omicron Aps, Roskilde, Denmark; 6 Department of Clinical Medicine, University of Copenhagen, Copenhagen, Denmark

## Abstract

**Background:**

Few randomized trials have evaluated the effect of postdischarge interventions for patients with liver cirrhosis. This study assessed the effects of a postdischarge intervention on readmissions and mortality in patients with decompensated liver cirrhosis.

**Methods:**

We conducted a randomized controlled trial at a specialized liver unit. Adult patients admitted with complications of liver cirrhosis were eligible for inclusion. Participants were allocated 1:1 to standard follow-up or a family-focused nurse-led postdischarge intervention between December 1, 2019, and October 31, 2021. The 6-month intervention consisted of a patient pamphlet, 3 home visits, and 3 follow-up telephone calls by a specialized liver nurse. The primary outcome was the number of readmissions due to liver cirrhosis.

**Results:**

Of the 110 included participants, 93% had alcohol as a primary etiology. We found no significant differences in effects in the primary outcomes such as time to first readmission, number of patients readmitted, and duration of readmissions or in the secondary outcomes like health-related quality of life and 6- and 12-month mortality. A post hoc exploratory analysis showed a significant reduction in nonattendance rates in the intervention group (RR: 0.28, 95% CI: 0.13–0.54, *p*=0.0004) and significantly fewer participants continuing to consume alcohol in the intervention group (*p*=0.003). After 12 months, the total number of readmissions (RR: 0.76, 95% CI: 0.59–0.96, *p*=0.02) and liver-related readmissions (RR: 0.55, 95% CI: 0.36–0.82, *p*=0.003) were reduced in the intervention group.

**Conclusions:**

A family-focused postdischarge nursing intervention had no significant effects on any of the primary or secondary outcomes. In a post hoc exploratory analysis, we found reduced 6-month nonattendance and alcohol consumption rates, as well as reduced 12-month readmission rates in the intervention group.

## INTRODUCTION

When patients present with decompensated liver cirrhosis, self-care and lifestyle changes are essential aspects of managing the disease.^1^ However, many patients with liver cirrhosis face considerable barriers to coping with their disease, and their self-care often proves inadequate.^2^ On average, patients with decompensated liver cirrhosis are admitted 3 times a year, and 20%–37% are readmitted <30 days after discharge.^3–5^ A lack of structure in outpatient care has been shown to indirectly cause repeated readmissions due to complications of cirrhosis,^6^ and it has been suggested that a closer and more structured postdischarge follow-up can prevent up to one-third of readmissions.^3,7^

Pulmonary and cardiac rehabilitation has shown significant effects as a secondary preventative strategy and is recommended as standard care.^8,9^ Nurse-assisted case-management follow-up interventions using telephone calls and home visits may be the most effective follow-up program for heart failure.^10^ Despite a clear need, little attention has been given to liver rehabilitation, and only a few randomized trials have evaluated the effects of nurse-assisted postdischarge interventions on readmission rates for patients with liver cirrhosis.^11^ Only 1 randomized pilot study tested a care management intervention involving home visits; it was limited by having only 20 participants in the control group and found no difference between the groups in their risk of death or liver-related hospital admissions.^12^ Family, including informal caregivers, are essential contributors when coping with liver cirrhosis.^1,13^ The conceptual framework of Family-Focused Nursing (FFN) has been used in several interventional studies and has demonstrable benefits for self-efficacy, social support, and coping with other chronic diseases.^14,15^ No randomized studies, and only a few clinical trials, for liver cirrhosis have included families and caregivers in postdischarge interventions.^16–18^

This randomized controlled trial aimed to assess the effects of a nurse-driven, family-focused, postdischarge intervention on readmissions and mortality in patients with decompensated liver cirrhosis and how it compares to standard follow-up in a specialized liver unit.

## METHODS

### Study design

This randomized controlled trial was investigator-initiated and registered at clinicaltrials.gov (NCT04158986) and approved by the Scientific Ethics Committees for the Capital Region of Denmark (H-19027459). The trial followed the Principles for Medical Research Involving Human Subjects^19^ and written consent was given in writing by all participants.

Patients were consecutively enrolled from the Gastro Unit, Medical Division, Copenhagen University Hospital Amager-Hvidovre, Denmark. Copenhagen University Hospital Amager-Hvidovre has a catchment area of 560,000 citizens, all living within a maximum distance of 12 miles from the hospital. In Denmark, all residents have a unique 10-digit social security number and benefit from universal tax–funded access to health care at general practitioners and hospitals.

The enrollment of participants took place during admission and was planned to last 19 months, starting on December 1, 2019.

Inclusion criteria required that participants were (i) adults (18 y or older); (ii) able to understand Danish; (iii) had been diagnosed with liver cirrhosis based on liver biopsy or findings in biochemistry, ultrasound, CT scan, liver elastography, or other relevant imaging; and (iv) had 1 or more liver-related complications leading to their admission.

Exclusion criteria were (i) when the diagnosis of liver cirrhosis was questioned with reasonable doubt, or the diagnosis of liver cirrhosis had been disproved; (ii) the presence of conditions or comorbidities where an independent rehabilitation program was offered; (iii) diagnosis of an active and invasive malignant disease; or (iv) cognitive deficits.

### Randomization

One-to-one randomization was performed by computer-generated shuffled numbers in blocks of 6 and delivered in opaque envelopes. The randomization was stratified according to family attendance during the intervention. Randomized sequence generation and allocation were blinded. The random allocation sequence was performed by the primary investigator. Participants randomized to the control group received standard postdischarge care. Participants randomized to the intervention group participated in a 6-month postdischarge intervention added to standard care.

### Standard care

All patients with liver cirrhosis at the Gastro Unit, Amager-Hvidovre Hospital, are routinely offered follow-up in an outpatient clinic by a team of hepatologists and liver nurses.

### Intervention

The intervention consisted of 3 elements added to standard care: (1) a patient pamphlet, (2) 3 home visits, and (3) 3 follow-up telephone calls, based on the person-centered and family-centered conceptual framework of FFN.^20^ FFN is a relational process in which nurses engage patients and possible families in nonhierarchical family-focused dialogues.^20,21^ The health care professional supports patients and families in managing the experienced challenges and assists patients in gaining a higher functional level or improved health based on the family’s own goals, wishes, and abilities.^20–22^ The intervention in this study is based on concepts from the practice-based Calgary Assessment Model (CFAM) and Calgary Intervention Model (CFIM). A thorough description of FFN is presented in the Supplemental Digital Content 1 (SDC 1, Conceptual framework of Family-Focused Nursing, http://links.lww.com/HC9/A857). A 3-day, FFN course, including theoretical and practical exercises, was required for the 2 project nurses involved in the study. Guidance and supervision for the project nurses were offered throughout the study period.

Before discharge, the participants received a pamphlet with information about preventative measures, early signs of decompensation, and relevant contact information. A description of the development and content of the pamphlet is presented in the Supplemental Digital Content (SDC 2, Development and content of patient pamphlet, http://links.lww.com/HC9/A858). Besides being a daily learning tool for the participant and family, the pamphlet was used as instructive material during home visits. The participants and possible family members or informal caregivers received a monthly home visit during the first 12 weeks after discharge by a project nurse specialized in the care of participants with chronic liver disease. The first home visit should be carried out within 3 weeks after discharge. The family members or informal caregivers selected by the participants were asked to attend these home visits, but it was not a demand. The intervention could take place at the hospital if requested by the participant and family. The home visits were based on family-focused therapeutic dialogues and included evidence-based information and guidance and helped to initiate contact with relevant municipal services such as home health care, physical exercise, social activities, medication dosage, alcohol rehabilitation, or financial guidance. The evidence-based information and guidance were provided based on the participant’s and family’s current problems or symptoms assessed during the therapeutic dialogues and by objective assessments during the home visits and were individualized based on the participant’s and family’s wishes, baseline knowledge, and receptiveness. At every visit, participants with alcohol as an etiology were asked about their current alcohol consumption and offered support in a nonjudgmental manner. It was emphasized that alcohol abstinence or reduction was highly recommended but that a relapse would not result in exclusion from the study or judgmental behavior from the project nurse. The project nurses were able to contact a designated physician with medicine-related questions and could initiate additional visits at the outpatient clinic. After the first 3 months, participants were followed up by telephone every month for 3 months; these calls included therapeutic dialogues, evidence-based information and guidance, and follow-up on the use of municipal services.

### Study outcomes

The primary outcome was the number of readmissions due to liver cirrhosis. Secondary outcomes were the time from discharge to the first readmission, the duration of readmissions due to liver cirrhosis, participants’ perceived health-related quality of life before and after the intervention, measured by the Chronic Liver Disease Questionnaire (CLDQ),^23^ and 6- and 12-month mortality. Outpatient nonattendance at the 6-month follow-up, alcohol consumption at the 6-month follow-up, and 12-month readmission rates were all subject to exploratory post hoc analysis.

In this study, readmission included any days in the hospital that followed continuously from the initial admission. We included 4 mutually exclusive readmission categories: liver-related readmissions, ascites readmissions, non-liver–related readmissions, and short readmissions. Liver-related readmissions included readmissions longer or equal to 24 hours that occurred due to liver cirrhosis including HE, complications due to ascites including infection, electrolyte disturbances, kidney failure, gastrointestinal bleeding, and reduced ability to take care of oneself due to poor general condition caused by liver cirrhosis. An ascites admission was defined as one where paracentesis was the only treatment. A non-liver–related admission included all readmissions longer than or equivalent to 24 hours caused by conditions or diseases with no direct relation to liver cirrhosis including fractures, alcohol detoxification, and psychiatric admissions. Short readmissions were any readmissions shorter than 24 hours, excluding ascites readmissions. In case of doubt about the distribution in the readmission categories, the research team hepatologists were consulted.

Data were collected through patient records and questionnaires. Data about alcohol consumption at baseline and follow-up were collected using questionnaires and assessment of patient records. At baseline, the participants were asked if they currently consumed alcohol. If yes, they were further asked about their weekly consumption based on the guidelines of the Danish Health Authority’s guidelines: moderate drinking (<7 units of alcohol weekly for women and <14 for men), heavy drinking (>7 units of alcohol weekly for women and >14 for men), or alcohol dependence. If no, they were asked about previous alcohol consumption and consumption levels. At follow-up, the participants were asked about their current consumption level as above. If a discrepancy between the results was found, the patient record was used. Data about weekly consumption of alcohol were collected solely by questionnaires.

### Statistical methods

Based on studies about admissions for cirrhosis,^3^ we expected to reduce admissions from 3 to 2 within 6 months. With a type 1 error of 5% and a power of 85%, 41 participants were needed in each group to achieve this reduction. Due to the high mortality and an expected dropout rate of 20%, we aimed to include 55 participants in each group.

R version 4.2 was used to analyze the data. Data for the number of participants readmitted per group and the number of deaths within the 6-month follow-up period were presented descriptively by group using frequencies and corresponding percentages. Group comparisons were carried out using the chi-square test, Poisson regression, or negative binomial regression. The time from discharge to the first readmission was analyzed using the Kaplan-Meier estimator. Groups were compared using the log-rank test. Participants who died during the study were handled in 2 different ways. In one analysis, participants were withdrawn from the study at the time of death. In another analysis, death was included as a readmission event. The duration of readmissions due to liver cirrhosis within 6 months was analyzed by a negative binomial regression to account for overdispersion. To analyze nonattendance events per participant in each group, we used Poisson regression with the logarithm of follow-up time as the offset. For each outcome, multiple regression analyses were conducted as secondary analyses and presented in addition to the primary results. In addition to the group variable, we adjusted for the covariates of gender, age group, MELD, and educational level. CLDQ data were presented descriptively by group and time (baseline and end-of-study). We calculated scores for the 6 domains and total scores and then compared these to the baseline. Group comparisons for the change from baseline were made using a *t* test. Data about alcohol consumption were collected at baseline and the end of the study and presented descriptively using frequencies and corresponding percentages. Shift tables were produced to show the changes in alcohol consumption. To check for randomness in the missing CLDQ and alcohol consumption values, we compared patient characteristics based on whether or not CLDQ or alcohol data were missing. To account for missing values, sensitivity analysis was conducted, where we applied the imputation method of carrying the baseline value forward when alcohol and CLDQ data were missing at the 6-month follow-up.

## RESULTS

### Participant flow

**FIGURE 1:**
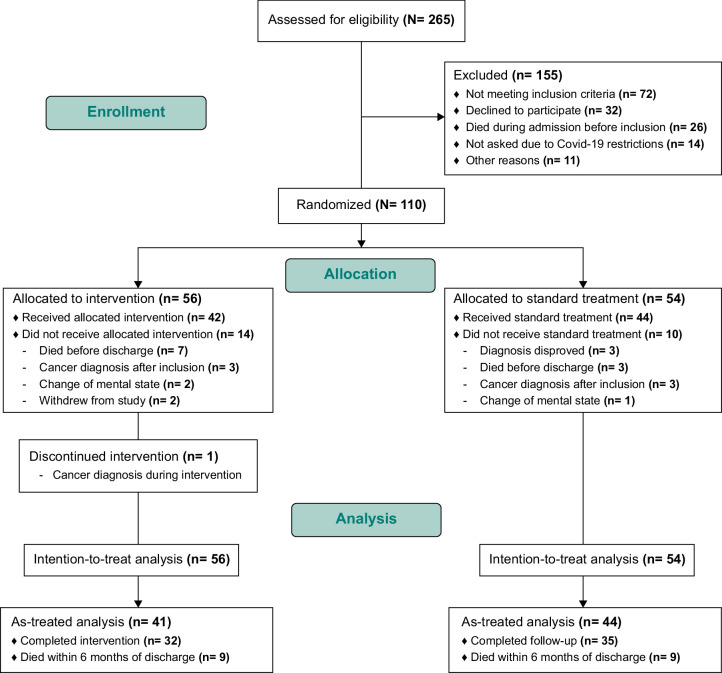
Trial flowchart of a family-focused, postdischarge intervention for patients with decompensated liver cirrhosis.

Participants were enrolled between December 1, 2019, and October 31, 2021. Due to Covid-19 lockdowns, the inclusion period was extended by 3 months. The trial ended when the sample size was reached. In total, 265 patients were assessed for eligibility, of whom 155 were excluded. Reasons for exclusion are presented in Figure 1. We included 110 participants who were allocated to the control group (n=54) or the intervention group (n=56). Ten participants in the control group and 13 in the intervention group were excluded due to death before discharge and exclusion criteria identified after inclusion, and 2 participants withdrew their consent, leaving 44 participants to receive standard treatment and 41 to receive the intervention (Figure 1). Intention-to-treat (ITT) analysis was performed on the 110 included participants, and complementary as-treated (AT) analysis was performed on the 85 participants who participated in the intervention.

### Participant characteristics

The baseline clinical and demographic characteristics of all 110 included participants are presented in Tables 1 and 2. Alcohol was the primary etiology (92.8%). MELD and Child-Pugh scores were comparable between groups, while imbalances were seen by chance between the control and intervention groups regarding time since diagnosis, educational, and income levels (Tables 1 and 2).

**TABLE 1:** Participants’ baseline clinical characteristics

	Control (N=54)	Intervention (N=56)	Overall (N=110)
Time since diagnosis, n (%)			
0–12 months	31 (54.9)	41 (73.2)	72 (64.5)
>12 months	23 (45.1)	15 (26.8)	38 (35.5)
Etiology, n (%)			
Alcohol	50 (92.5)	52 (92.8)	102 (92.8)
Methotrexate	2 (3.7)	0 (0)	2 (1.8)
Primary biliary cholangitis	0 (0)	2 (3.6)	2 (1.8)
Autoimmune hepatitis	1 (1.9)	1 (1.8)	2 (1.8)
NASH	1 (1.9)	1 (1.8)	2 (1.8)
No. comorbidities, n (%)^a^			
0–1	23 (42.6)	24 (42.9)	47 (42.7)
2–3	24 (44.4)	25 (44.6)	49 (44.6)
>4	7 (13.0)	7 (12.5)	14 (12.7)
MELD score			
Mean [min, max]	16.5 [10, 35]	16.0 [10, 29]	16.2 [10, 35]
Child-Pugh score, n (%)			
B (8–9)	29 (53.7)	26 (46.4)	55 (50.0)
C (>9)	25 (46.3)	30 (53.6)	55 (50.0)
Alcohol consumption level at inclusion, n (%)^b^			
No	17 (31.5)	22 (39.3)	38 (34.5)
Yes	37 (68.5)	34 (60.7)	72 (59.5)
Main reason for initial admission, n (%)			
Ascites	21 (38.9)	29 (51.8)	50 (45.4)
HE	9 (16.7)	9 (16.1)	18 (16.4)
Variceal bleeding	8 (14.8)	9 (16.1)	17 (15.5)
Electrolyte disturbance	9 (16.7)	5 (8.9)	14 (12.7)
Icterus	6 (11.1)	2 (3.6)	8 (7.3)
Bacterial peritonitis	1 (1.8)	1 (1.8)	2 (1.8)
Anemia	0 (0)	1 (1.8)	1 (0.9)

a Defined as the simultaneous presence of current diseases or medical conditions in addition to liver cirrhosis, not including any direct complications to liver cirrhosis.

b Defined as alcohol consumption level in the month before admission.

**TABLE 2:** Participants’ demographic characteristics

	Control (N=54)	Intervention (N=56)	Overall (N=110)
Age			
Median [min, max]	61.2 [38, 80]	61.3 [37, 81]	61.7 [37, 81]
Age group, n (%)			
36–45	3 (5.6)	2 (3.6)	5 (4.5)
46–55	14 (25.9)	13 (23.2)	27 (24.5)
56–65	15 (27.8)	18 (32.1)	33 (30.0)
66–75	18 (33.3)	20 (35.7)	38 (34.5)
76–85	4 (7.4)	3 (5.4)	7 (6.5)
Gender, n (%)			
Female	20 (37.0)	25 (44.6)	45 (40.9)
Male	34 (63.0)	31 (55.4)	65 (59.1)
Marital status, n (%)			
Civil partnership	7 (13.0)	7 (12.5)	14 (12.1)
Divorced	14 (25.9)	19 (33.9)	33 (29.0)
Married	16 (29.6)	16 (28.6)	32 (29.9)
Unmarried	11 (20.4)	10 (17.9)	21 (19.7)
Widowed	6 (11.1)	4 (7.1)	10 (9.3)
Education level, n (%)			
Primary school	18 (33.3)	10 (17.9)	28 (25.5)
Special worker education	1 (1.9)	1 (1.8)	2 (1.8)
Apprenticeship training	12 (22.2)	22 (39.3)	34 (30.9)
Shorter theoretical education (1–3 y)	13 (24.1)	10 (17.9)	23 (20.9)
Longer theoretical education (>3 y)	7 (12.9)	7 (12.5)	14 (12.7)
Academic education	3 (5.6)	6 (10.7)	9 (8.2)
Annual gross income (DKK/y), n (%)			
<100,000–250,000^a^	36 (66.7)	36 (64.3)	72 (65.5)
250,000–550,000^b^	11 (20.4)	14 (25.0)	25 (22.7)
550,000–>700,000^c^	0 (0)	3 (5.4)	3 (2.7)
Missing	7 (12.9)	3 (5.4)	10 (9.1)

a Approximately 14,400–36,100 USD.

b Approximately 36,100–79,500 USD.

c Approximately 73,600–101,100 USD.

A process analysis of intervention mechanisms showed that the home visits had a median duration of 60 minutes (range: 30–90 min), and the follow-up telephone calls lasted 15–30 minutes. The project nurses facilitated medical interventions for 27 participants consisting of medicine titration (23 interventions), booking of blood tests (17 interventions), booking of elective paracentesis (7 interventions), referral to general practitioner (2 interventions), and facilitation of hospital admissions (2 interventions). Twenty-seven participants were referred by the project nurse to municipal services.

### Primary endpoints

#### Time from discharge to the first readmission

**FIGURE 2:**
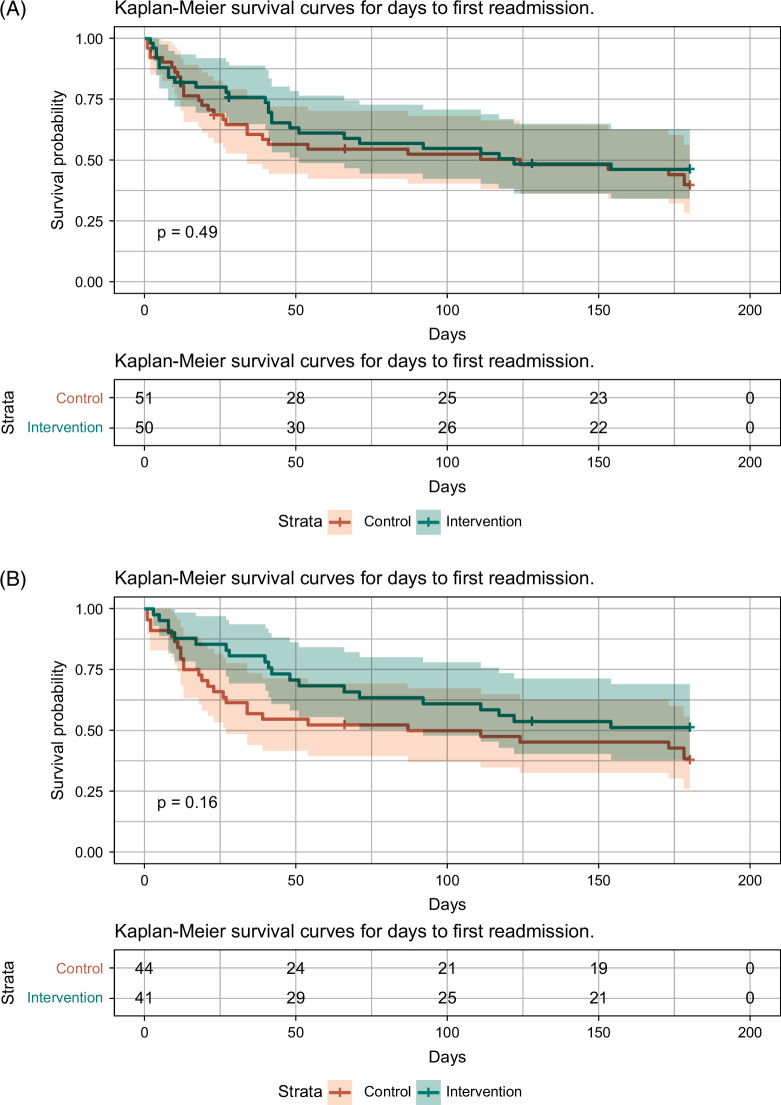
(A) Kaplan-Meier curves for the number of days until the first readmission in the intention-to-treat population. (B) Kaplan-Meier curves for the number of days until the first readmission in the as-treated population.

Time from discharge to the first readmission with death as an observed event was longer in the intervention group than in the control group, albeit nonsignificant in both ITT analysis (*p*=0.49) (Figure 2A) and AT analysis (*p*=0.16) (Figure 2B).

#### Six-month readmissions

We found no significant evidence of a difference between the 6-month readmission rates in the 2 groups.

Both ITT and AT analyses showed positive but nonsignificant differences in the intervention group compared to the control group at the 6-month follow-up, both regarding the total number of readmissions and number of liver-related readmissions (Table 3). The same was the case for the number of participants readmitted. Both ITT and AT analyses showed a nonsignificant decrease in the number of participants readmitted for any cause and the number of participants with liver-related readmission in the intervention group compared to the control group (Table 3).

**TABLE 3:** Readmission rates at 6- and 12-month follow-up

	Intention-to-treat analysis			As-treated analysis		
	Control (N=54)	Intervention (N=56)	*p*	Control (N=44)	Intervention (N=41)	*p*
No. participants readmitted within 6 months						
Number of participants with a liver-related readmission	28	24	0.553	27	19	0.165
Number of participants with a non-liver–related readmission	7	8	0.779	5	6	0.905
Number of participants with an ascites readmission	4	4	0.953	3	3	0.928
Number of participants with a short (<24 h) readmission	10	10	0.920	9	9	0.865
Total number of participants readmitted	35	29	0.325	32	23	0.195
No. readmissions at 6-month follow-up						
Liver-related readmissions	53	43	0.403	51	37	0.126
Non-liver–related readmissions	15	10	0.334	9	6	0.605
Ascites admissions^a^	6	12	—	3	10	—
Short (<24 h) readmissions	13	14	0.772	12	13	0.860
Total number of readmissions	87	79	0.881	75	66	0.574
No. participants readmitted within 12 months						
Number of participants with a liver-related readmission	35	28	0.141	34	23	0.038
Number of participants with a non-liver–related readmission	14	10	0.233	12	7	0.154
Number of participants with an ascites readmission	5	4	0.774	4	3	0.766
Number of participants with a short (<24 h) readmission	21	16	0.471	20	15	0.504
Total number of participants readmitted	39	32	0.310	36	26	0.093
No. of readmissions at 12-month follow-up						
Liver-related readmissions	89	58	0.003	87	51	0.026
Non-liver–related readmissions	30	16	0.071	20	9	0.058
Ascites admissions^a^	8	13	—	5	10	—
Short (<24 h) readmissions	30	25	0.719	29	22	0.391
Total number of readmissions	157	112	0.024	141	92	0.005

a Too few non-zero observations to get reliable estimates of the risk.

#### Duration of readmissions due to liver cirrhosis within 6 months

ITT analysis showed a nonsignificant decrease in the length of first readmission with a mean length of 2.67 (SD=4.0) in the intervention group and 4.36 (SD=5.36) in the control group, an estimated RR of 0.613 (95% CI: 0.301–1.247, *p*=0.173). AT analysis also showed a nonsignificant 36.3% decrease in the expected number of days admitted during the first liver-related admission in the intervention group with 2.8 days (SD=4.1) compared to the control group with 4.4 days (SD=5.4) (RR: 0.637, 95% CI: 0.312–1.307, *p*=0.214).

Adjusting for the covariates of gender, age group, MELD score, and educational level did not alter the conclusions above.

### Secondary endpoints

#### Health-related quality of life

Analysis for randomness in the missing CLDQ values showed a significant difference between nonmissing and missing groups in the gender characteristic (Supplemental Tables S1–S2, http://links.lww.com/HC9/A859). After conducting a sensitivity analysis, we saw no significant change in the total CLDQ results between the 2 groups at the end of the study (Supplemental Figure S1, http://links.lww.com/HC9/A860). We saw no significant change in the total score (*p*=0.280) or in the 6 domains individually: fatigue (*p*=0.346), emotional function (*p*=0.592), abdominal symptoms (*p*=0.267), systemic symptoms (*p*=0.475), worry (*p*=0.805), and activity (*p*=0.125).

#### Six- and 12-month mortality

ITT analysis showed that at the 6-month follow-up, 14 participants had died in the control group and 21 in the intervention group (*p*=0.193), while AT analysis showed that 9 participants in each group had died (*p*=0.912). By the 12-month follow-up, ITT analysis showed that 19 participants had died in the control group and 25 in the intervention group (*p*=0.311), while AT analysis showed 13 participants in the intervention group and 14 in the control group had died (*p*=0.931) (Supplemental Table S7, http://links.lww.com/HC9/A861).

### Post hoc exploratory analysis

#### Nonattendance

**FIGURE 3:**
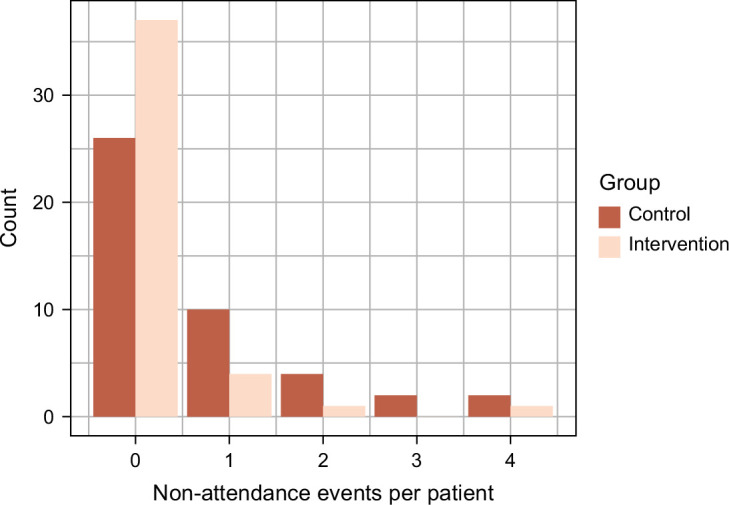
The total number of nonattendance events per participant in each group in the intention-to-treat population.

In a post hoc exploratory analysis, we saw a significant improvement in the attendance rate for outpatient visits at the 6-month follow-up in the intervention group (Figure 3).

ITT analysis showed that 6 participants (10.7%) in the intervention group did not appear for one or more liver-related outpatient visits compared to 21 (38.9%) in the control group (*p*=0.0011). In the intervention group, the participants had 10 nonattendances, compared to 37 in the control group, a RR of 0.280, 95% CI: (0.132–0.542) (*p*=0.00036). AT analysis also showed a significant reduction with 5 participants (12.2%) in the intervention group with one or more liver-related nonattendances compared to 18 (40.9%) in the control group (*p*=0.003). In the intervention group, AT analysis showed that participants did not appear for 6 liver-related outpatient visits, compared to 32 in the control group, showing an expected rate of nonattendance of RR: 0.181, 95% CI (0.068–0.403), indicating a significant decrease of 81.4% between the 2 groups (*p*=0.00015).

We found no change in these results after adjusting for the covariates of gender, age group, MELD score, and educational level.

#### Alcohol consumption

The analysis of alcohol consumption data revealed expected missing alcohol consumption values, especially weekly consumption values, which were collected solely by questionnaires. Analysis for randomness in the missing alcohol consumption values showed a significant difference in several characteristics (Supplemental Tables S3–S6, http://links.lww.com/HC9/A859). We conducted a sensitivity analysis, which showed a significant change in alcohol consumption between the 2 groups in both ITT and AT analyses (Supplemental Table S8, http://links.lww.com/HC9/A862). ITT analysis showed 35 (62.5%) participants in the intervention group consuming alcohol at baseline and 17 (30.4%) at the 6-month follow-up, compared to 40 (74.1%) participants consuming alcohol in the control group at baseline and 36 (66.7%) at follow-up (*p*≤0.001) (Supplemental Table S8, http://links.lww.com/HC9/A862). We saw no change in this intervention effect after correcting for baseline consumption.

#### 12-month readmissions

In light of the positive effects on alcohol consumption and adherence to visits in outpatient clinics in the intervention group, an exploratory analysis was performed on 12-month readmission rates.

ITT analysis of the total number of admissions across 12 months showed a significant reduction in the total number of admissions, with 112 admissions in the intervention group (mean number of admissions per participant of 2.29) versus 157 in the control group (mean number of admissions per participant of 3.16). The estimated RR of readmission was 0.757, 95% CI (0.593–0.962) for the intervention group compared to the control group (*p*=0.024) (Table 3). AT analysis also showed a significant 31.8% decrease in admissions in the intervention group compared to the control group, with an estimated RR of readmission of 0.682, 95% CI (0.522–0.887) (*p*=0.005) (Table 3).

ITT analysis further showed a significant difference in the number of liver-related readmissions, with 58 in the intervention group compared to 89 in the control group. The estimated RR of readmission was 0.551, 95% CI (0.364–0.817) in the intervention group compared to the control group (*p*=0.003) (Table 3). AT analysis also showed a significant 29.3% decrease in liver-related readmissions between groups with an estimated RR of readmission of 0.707, 95% CI (0.518–0.957) in the intervention group compared to the control group (*p*=0.026) (Table 3).

Both ITT and AT analyses showed a nonsignificant decrease in the number of participants with a readmission of any cause in the intervention group compared to the control group (Table 3). ITT analysis further showed a nonsignificant result regarding the number of participants with a liver-related readmission (Table 3). AT analysis did though show a significant decrease in the number of participants with a liver-related readmission in the intervention group, with 23 participants readmitted compared to 34 in the control group (*p*=0.038) (Table 3).

We saw no significant difference between groups regarding ascites readmissions and short readmissions in either ITT or AT analysis (Table 3).

## DISCUSSION

In this randomized, family-focused, nurse-led, postdischarge intervention, mainly among patients with alcoholic liver cirrhosis, we found improvements but no statistically significant differences between groups in the primary outcomes such as time to first readmission, number of patients readmitted, and duration of readmissions. We saw no changes in the secondary outcomes HRQoL and 6- and 12-month mortality. A post hoc exploratory analysis of attendance for outpatient visits showed that significantly fewer patients missed liver-related visits in the intervention group compared to the control group. Furthermore, we saw significantly more abstinent participants at the 6-month follow-up in the intervention group than in the control group. The positive effects on attendance rates and alcohol consumption in the intervention group prompted us to analyze 12-month readmission data, with the hypothesis that improved attendance rates and alcohol abstinence might improve the readmission rates on a longer-term basis. The 12-month exploratory analyses showed a significant difference between groups in the number of participants with 1 or more liver-related readmissions and a significant difference between the 2 groups in the total number of admissions and liver-related readmissions in favor of the intervention group. We recognize that the significant findings in this study are exploratory and should be considered hypothesis-generating.

Only 2 properly equipped, nurse-assisted randomized clinical trials have presented mortality and readmissions as intervention outcomes.^11^ A standardized hospital follow-up program by Morando et al^24^ showed lower all-cause mortality and a significant reduction in 30-day readmissions, days of hospitalizations among patients with liver cirrhosis and ascites, and 12-month readmission rates in the intervention group. Morando and colleagues included participants with ascites as a complication of liver cirrhosis; in contrast, in our study, we included patients with all types of complications due to liver cirrhosis. Furthermore, in the study by Morando et al,^24^ 37% of the participants had alcohol as the etiology compared to 93% of the participants in our study. Our study was conducted in a highly specialized liver unit where patients, as standard care, are followed by specialized hepatologists and liver nurses, that is, care comparable to the intervention tested by Morando et al.^24^ The differences between the participants and the close similarity between our standard care group and the intervention group in the study by Morando et al^24^ might explain the differences in readmission results between the studies. A pilot study by Wigg and colleagues conducted an individualized case-management intervention, including home visits and weekly telephone follow-ups. That study showed no significant difference in mortality or hospital readmissions.^12^ The small sample size and differences in disease etiology between the groups could explain the lack of significant results.^12^ Three ongoing studies will hopefully provide further evidence concerning nurse-assisted, individualized management of cirrhosis.^25–27^

A lack of consistent results about readmissions and mortality among postdischarge intervention patients with decompensated liver cirrhosis^11,28^ and the absence of a significant improvement in 6-month readmissions, as well as the high mortality rate in this study, could indicate that readmissions and mortality are not suitable endpoints for patients with advanced liver cirrhosis. According to the Supportive and Palliative Care Indicators Tool (SPICT),^29^ a majority of the participants in this study would be eligible for palliative care. There is currently insufficient palliative care offered to patients with liver cirrhosis,^30^ and the implementation of palliative care programs is recommended.^31^ Clinical trials combining palliative care and potentially life-sustaining therapy are warranted.^32^

In an exploratory analysis, we saw a significantly higher attendance rate in the intervention group than in the control group. A similar finding was made by Wigg et al.^12^ In both of these studies, an individualized, person-centered and family-centered approach was adopted for the intervention. Person-centered and family-centered care focuses on respect and dignity, shared decision-making, participation, and collaboration.^33^ Interventions with a person-centered and family-centered approach, including family-focused interventions, have been shown to improve self-efficacy and social support among patients with coronary heart syndrome.^14,15^ Self-efficacy is considered key for self-care behavior across several other chronic diseases and conditions,^34,35^ and increased self-efficacy could explain the positive results on both attendance and alcohol consumption in this study. Person-centered and family-centered approaches, including family-focused interventions, should be considered in future interventional studies of patients with decompensated liver cirrhosis.

Our findings suggest that we should increase our focus on differentiated treatment paths for patients with liver cirrhosis according to the severity of their disease and individual wishes and needs. We should increase our efforts to identify patients who could benefit from person-centered and family-centered follow-up programs and seek to offer palliative care to patients and families who need it.

The strengths of this randomized controlled study were that the intervention was well-defined, that the predefined sample size was reached, and that the intervention it tested proved safe and feasible. There were also some limitations. This was a single-center study. The baseline imbalances between the control and intervention groups despite randomization could have caused bias in the intervention effect estimate. The study suffered some information bias due to partially missing data, and recall bias was a risk in the sociodemographic and CLDQ questionnaires, which rely on participants’ memory and truthfulness. Furthermore, alcohol biomarkers or validated questionnaires for alcohol consumption were not used in this study which could lead to misclassification bias. Due to the large number of missing CLDQ and weekly alcohol consumption values, multiple imputations could have been used to account for missing data. The intervention was intended to include patients with liver cirrhosis from all causes, but 93% of its participants had alcohol as their primary etiology—a product of the study catchment area. Therefore, our results cannot be generalized to other causes of liver diseases.

In conclusion, this study showed no significant difference between study groups in the primary outcomes such as time to first readmission, number of patients readmitted, and duration of readmissions or in the secondary outcomes like HRQoL and mortality. A post hoc exploratory analysis showed reduced 6-month nonattendance rates and alcohol consumption in the intervention group, and a following analysis of 12-month readmission data showed a significant reduction in the total number of admissions and the number of liver-related readmissions. Future studies further exploring these findings are warranted.

## Supplementary Material

SUPPLEMENTARY MATERIAL
