# Supplementary material for: A randomized controlled trial of a postdischarge nursing intervention for patients with decompensated cirrhosis
Source: Hepatol Commun. 2024 Apr 26;8(5):e0418. doi: 10.1097/HC9.0000000000000418 (PMC12333763; doi:10.1097/HC9.0000000000000418)
Supplement: SUPPLEMENTARY MATERIAL [file hc9-8-e0418-s001.docx]

**SDC 1. The conceptual framework of Family-Focused Nursing.**

The conceptual framework of Family-Focused Nursing (FFN) was originally developed by Wright and Leahey (1). FFN is based on the clinical practice models the Calgary Assessment Model (CFAM) and Calgary Intervention Model (CFIM) and the Illness Belief Model (IBM) (1, 2). CFAM and CFIM are theoretical tools grounded in clinical practice forming a framework for Family-Focused therapeutic dialogues between health professionals, patients, and families with the purpose to create a context for change. In FFN, family is defined by the patient and is not necessarily marital or blood-related (1). CFAM and CFIM can be used in dialogues with only the patients present or in dialogues with a family (3). Elements of the tools can be used during brief meetings with patients or families, and more extensive use of the tools can be utilized in a more structured setting. CFAM and CFIM are always delivered to the patient and family in a non-hierarchical context and caring relationship targeted to their unique needs (1, 3).

CFAM and CFIM are structured with an assessment phase (CFAM) and intervention phase (CFIM) utilized in a dynamic and overlapping structure. The IBM is embedded in the CFAM and CFIM, recognizing that patients, families, and healthcare professionals all have beliefs that affect how we see the world and live our lives (2, 4). The IBM states that it is not the illness itself that causes the greatest suffering but rather the beliefs about the illness. Illness beliefs can be both facilitating and constraining and have a great influence on illness experience and relationships with both family members and healthcare professionals. Constraining beliefs should be explored, challenged, and tried to change, and facilitating beliefs should be acknowledged and strengthened (4).

During the assessment phase, CFAM enables healthcare professionals to assess family strengths, resources, and problems concerning their illness through targeted questions. The healthcare professionals assess the patient and families according to problem identification, family interaction according to the health and illness-related challenges, and attempted solutions to solve the challenges and goals. Genograms and ecomaps can be drawn in collaboration with patients and families to determine the family structure, development, and function. The genograms and ecomaps can be utilized as visual tools to enhance patients’ and families’ understanding of the family structure of their health and illness-related challenges.

CFIM consists of specific interventive questions and commendations, which the healthcare professionals can offer to the families. Within the CFIM the aim is to modify the patient and family’s beliefs about a problem or illness and to facilitate change and by that reduce suffering. The interventive questions are created by the healthcare professionals from eight interventions comprising; Encourage a health narrative; Give time; Propose/assign rituals; Emphasize family support; Recognize family and individual strengths, Encourage family members to be caregivers and offer support; Validate and normalize emotional responses; and Offer information and Opinions (1). The interventions are offered through a systematic approach that focuses on lineal, circular, strategic, and reflexive questions originally proposed by psychologist Karl Tomm (5).

FFN and CFAM/CFIM have been utilized in several interventional studies across several diseases and illnesses and have shown to be feasible and have shown positive effects on caregiver burden, HRQoL, satisfaction with health care services, perceived emotional and cognitive support, coping patterns and self-efficacy (6-9).

REFERENCES

1. Shajan Z, Snell D. Wright & Leahey's Nurses and Families: A Guide to Family Assessment and Intervention: F.A. Davis Company; 2019.

2. Bell JM, Wright LM. The Illness Beliefs Model: advancing practice knowledge about illness beliefs, family healing, and family interventions. J Fam Nurs. 2015;21(2):179-85.

3. Bell JM. Family Systems Nursing: re-examined. J Fam Nurs. 2009;15(2):123-9.

4. Bell LWJ. Beliefs and Illness: A Model for Healing: 4th Floor Press; 2009. 400 p.

5. Tomm K. Interventive interviewing: Part III. Intending to ask lineal, circular, strategic, or reflexive questions? Fam Process. 1988;27(1):1-15.

6. Petursdottir AB, Svavarsdottir EK. The effectivness of a strengths-oriented therapeutic conversation intervention on perceived support, well-being and burden among family caregivers in palliative home-care. J Adv Nurs. 2019;75(11):3018-31.

7. Svavarsdottir EK, Kamban SW, Konradsdottir E, Sigurdardottir AO. The Impact of Family Strengths Oriented Therapeutic Conversations on Parents of Children with a New Chronic Illness Diagnosis. J Fam Nurs. 2020;26(3):269-81.

8. Østergaard B, Mahrer-Imhof R, Shamali M, Nørgaard B, Jeune B, Pedersen KS, et al. Effect of family nursing therapeutic conversations on patients with heart failure and their family members: Secondary outcomes of a randomised multicentre trial. J Clin Nurs. 2021;30(5-6):742-56.

9. Petursdottir AB, Haraldsdottir E, Svavarsdottir EK. The impact of implementing an educational intervention to enhance a family-oriented approach in specialised palliative home care: A quasi-experimental study. Scand J Caring Sci. 2019;33(2):342-50.
