# Supplementary material for: A randomized controlled trial of a postdischarge nursing intervention for patients with decompensated cirrhosis
Source: Hepatol Commun. 2024 Apr 26;8(5):e0418. doi: 10.1097/HC9.0000000000000418 (PMC12333763; doi:10.1097/HC9.0000000000000418)
Supplement: SUPPLEMENTARY MATERIAL [file hc9-8-e0418-s002.docx]

**SDC 2. Development and content of patient pamphlet.**

The patient pamphlet was developed based on evidence-based knowledge of disease complications and preventative measures, and the contact details for relevant hospital wards. The pamphlet was pre-tested by two hepatologists and three specialized liver nurses, three patients admitted with decompensated liver cirrhosis, and three family members of patients with decompensated liver cirrhosis. The physicians and nurses were asked to determine if the content was sufficient and relevant. Patients and families were asked to determine if the content was understandable and of interest. After the pre-testing, changes were made to the pamphlet’s content, structure, and wording, and was approved by the ward management.

The pamphlet contained simple information regarding the risks and consequences of sarcopenia and nutritional advice including the positive effect of frequent meals, examples of protein-rich foods and bedtime snacks, and possible sodium restriction, nutritional- and vitamin supplements (1). Guidance regarding nutrition and physical exercise during the home visits was individualized based on the patient's and family’s current nutritional status, habits, and wishes. Furthermore, the pamphlet included a recommendation for alcohol abstinence and advice on talking to the project nurses if the patients had a wish for alcohol rehabilitation. Information regarding ascites, varices, and hepatic encephalopathy included a description of signs of accumulation of ascites, the grades- and early signs of hepatic encephalopathy, and signs of variceal bleeding. For all three complications, the pamphlet contained advice on preventive behaviour, medication guidance, and guidance on when and how to contact the general practitioner, home health care, hospital, or an ambulance, and the importance of compliance with both medication and examinations.

**REFERENCES**

1. Kamran U, Towey J, Khanna A, Chauhan A, Rajoriya N, Holt A. Nutrition in alcohol-related liver disease: Physiopathology and management. World J Gastroenterol. 2020;26(22):2916-30.
