# Supplementary material for: A randomized controlled trial of a postdischarge nursing intervention for patients with decompensated cirrhosis
Source: Hepatol Commun. 2024 Apr 26;8(5):e0418. doi: 10.1097/HC9.0000000000000418 (PMC12333763; doi:10.1097/HC9.0000000000000418)
Supplement: SUPPLEMENTARY MATERIAL [file hc9-8-e0418-s003.docx]

**SDC 3. Participant characteristics split by missing/non-missing CLDC and alcohol consumption values at 6-month follow-up**

**Table 1.** Participants’ demographic characteristics split by missing/non-missing CLDQ values at six-month follow-up

|  | **Missing**  **(N=64)** | **Non-missing**  **(N=46)** | **Total**  **(N=110)** | ***p-*value** |
| --- | --- | --- | --- | --- |
| **Age group** |  |  |  | 0.068 |
| 36-45 | 0 | 5 (10.9%) | 5 (4.5%) |  |
| 46-55 | 18 (28.1%) | 10 (21.7%) | 28 (25.5%) |  |
| 56-65 | 18 (28.1%) | 16 (34.8%) | 34 (30.9%) |  |
| 66-75 | 23 (35.9%) | 13 (28.3%) | 36 (32.7%) |  |
| 76-85 | 5 (7.8%) | 2 (4.3%) | 7 (6.4%) |  |
| Missing | 0 | 0 | 0 |  |
| **Gender** |  |  |  | 0.002 |
| Female | 41 (64.1%) | 16 (34.8%) | 57 (51.8%) |  |
| Male | 23 (35.9%) | 30 (65.2%) | 53 (48.2%) |  |
| Missing | 0 | 0 | 0 |  |
| **Marital status** |  |  |  | 0.241 |
| Civil partnership | 11 (17.2%) | 3 (6.5%) | 14 (12.7%) |  |
| Divorced | 22 (34.4%) | 11 (23.9%) | 33 (30.0%) |  |
| Married | 16 (25.0%) | 16 (34.8%) | 32 (29.1%) |  |
| Unmarried | 10 (15.6%) | 11 (23.9%) | 21 (19.1%) |  |
| Widowed | 5 (7.8%) | 5 (10.9%) | 10 (9.1%) |  |
| Missing | 0 | 0 | 0 |  |
| **Educational level** |  |  |  | 0.569 |
| Primary school | 14 (21.9%) | 12 (26.1%) | 26 (23.6%) |  |
| Special worker education | 2 (3.1%) | 1 (2.2%) | 3 (2.7%) |  |
| Apprenticeship training | 18 (28.1%) | 16 (34.8%) | 34 (30.9%) |  |
| Shorter theoretical education (1-3 years) | 16 (25.0%) | 6 (13.0%) | 22 (20.0%) |  |
| Longer theoretical education (>3 years) | 8 (12.5%) | 7 (15.2%) | 15 (13.6%) |  |
| Academic education | 5 (7.8%) | 4 (8.7%) | 9 (8.2%) |  |
| Missing | 1 (1.6%) | 0 | 1 (0.9%) |  |
| **Yearly income DKK (DKK/year)** |  |  |  | 0.386 |
| 100.000 - 250.000* | 44 (68.8%) | 26 (56.5%) | 70 (63.6%) |  |
| 250.000 - 550.000** | 11 (17.2%) | 14 (30.4%) | 25 (22.7%) |  |
| 550.000 - ­ >700.000*** | 1 (1.5%) | 2 (4.3%) | 3 (2.7%) |  |
| Missing | 8 (12.5%) | 4 (8.7%) | 12 (10.9) |  |

**Table 2.** Participants’ clinical characteristics split by missing/non-missing CLDQ values at six-month follow-up

|  | **Missing**  **(n=64)** | **Non-missing**  **(n=46)** | **Total**  **(N=110)** | ***p*-value** |
| --- | --- | --- | --- | --- |
| **Time since diagnosis** |  |  |  | 0.762 |
| < 6 months | 41 (64.1%) | 32 (69.6%) | 73 (66.4%) |  |
| > 12 months | 23 (35.9%) | 14 (30.4%) | 37 (33.6%) |  |
| Missing | 0 | 0 | 0 |  |
| **Etiology** |  |  |  | 0.800 |
| Alcohol | 58 (90.6%) | 43 (93.5%) | 101 (91.8%) |  |
| Methotrexate | 1 (1.6%) | 1 (2.2%) | 2 (1.8%) |  |
| PBC | 2 (3.1%) | 0 | 2 (1.8%) |  |
| AIH | 1 (1.6%) | 1 (2.2%) | 2 (1.8%) |  |
| NASH | 1 (1.6%) | 1 (2.2%) | 2 (1.8%) |  |
| Missing | 1 (1.6%) | 0 (0.0%) | 1 (0.9%) |  |
| **Number of comorbidities** |  |  |  |  |
| 0-1 | 23 (35.9%) | 24 (52.2%) | 47 (42.7%) |  |
| 2-3 | 33 (51.6%) | 17 (36.9%) | 50 (45.5%) |  |
| >4 | 8 (12.5%) | 5 (10.9%) | 13 (11.8%) |  |
| Missing | 0 | 0 | 0 |  |
| **Child-Pugh** |  |  |  | 0.579 |
| B (8-9) | 29 (45.3%) | 24 (52.2%) | 53 (48.2%) |  |
| C (>9) | 33 (51.6%) | 22 (47.8%) | 55 (50.0%) |  |
| Missing | 2 (3.1%) | 0 | 2 (1.8%) |  |
| **Alcohol consumption at inclusion** |  |  |  | 0.357 |
| No | 32 (50.0%) | 20 (43.5%) | 52 (47.3%) |  |
| Yes | 29 (44.3%) | 26 (56.5%) | 55 (50.0%) |  |
| Missing | 3 (4.7%) | 0 | 3 (2.7%) |  |
| **Main reason for admission** |  |  |  | 0.528 |
| Ascites | 34 (53.1%) | 19 (41.3%) | 53 (48.2%) |  |
| HE | 11 (17.2%) | 6 (13.0%) | 17 (15.5%) |  |
| Variceal bleeding | 7 (10.9%) | 9 (19.6%) | 16 (14.5%) |  |
| Electrolyte disturbance | 6 (9.4%) | 6 (13.0%) | 12 (10.9%) |  |
| Icterus | 4 (6.3%) | 5 (10.9%) | 9 (8.2%) |  |
| Bacterial peritonitis | 1 (1.6%) | 0 (0.0%) | 1 (0.9%) |  |
| Anemia | 0 (0.0%) | 1 (2.2%) | 1 (0.9%) |  |
| Missing | 1 (1.6) | 0 | 1 (0.9) |  |

**Table 3.** Participants’ demographic characteristics split by missing/non-missing alcohol consumption values at six-month follow-up

|  | **Missing**  **(n=10)** | **Non-missing (n=100)** | **Total**  **(N=110)** | ***p-*value** |
| --- | --- | --- | --- | --- |
| **Age group** |  |  |  | 0.611 |
| 36-45 | 0 (0.0%) | 5 (5.0%) | 5 (4.5%) |  |
| 46-55 | 1 (10.0%) | 26 (26.0%) | 27 (24.5%) |  |
| 56-65 | 3 (30.0%) | 32 (32.0%) | 35 (31.8%) |  |
| 66-75 | 5 (50.0%) | 31 (31.0%) | 36 (32.7%) |  |
| 76-85 | 1 (10.0%) | 6 (6.0%) | 7 (6.4%) |  |
| Missing | 0 | 0 | 0 |  |
| **Gender** |  |  |  | 0.001 |
| female | 10 (100.0%) | 47 (47.0%) | 57 (51.8%) |  |
| male | 0 (0.0%) | 53 (53.0%) | 53 (48.2%) |  |
| Missing | 0 | 0 | 0 |  |
| **Marital status** |  |  |  | 0.090 |
| Civil partnership | 3 (30.0%) | 11 (11.0%) | 14 (12.7%) |  |
| Divorced | 4 (40.0%) | 28 (28.0%) | 32 (29.1%) |  |
| Married | 1 (10.0%) | 31 (31.0%) | 32 (29.1%) |  |
| Unmarried | 0 (0.0%) | 22 (22.0%) | 22 (20.0%) |  |
| Widowed | 2 (20.0%) | 8 (8.0%) | 10 (9.1%) |  |
| Missing | 0 | 0 | 0 |  |
| **Educational level** |  |  |  | 0.217 |
| Primary school | 0 (0.0%) | 26 (26.0%) | 26 (23.6%) |  |
| Special worker education | 0 (0.0%) | 2 (2.0%) | 2 (1.8%) |  |
| Apprenticeship training | 4 (40.0%) | 31 (31.0%) | 35 (31.8%) |  |
| Shorter theoretical education (1-3 years) | 4 (40.0%) | 18 (18.0%) | 22 (20.0%) |  |
| Longer theoretical education (>3 years) | 1 (10.0%) | 14 (14.0%) | 15 (13.6%) |  |
| Academic education | 0 (0.0%) | 9 (9.0%) | 9 (8.2%) |  |
| Missing | 1 (10.0%) | 0 | 1 (0.9%) |  |
| **Yearly income DKK (DKK/year)** |  |  |  | 0.837 |
| 100.000 - 250.000* | 7 (70.0%) | 63 (63.0%) | 70 (63.6%) |  |
| >700.000 | 0 (0.0%) | 2 (2.0%) | 2 (1.8%) |  |
| 250.000 - 550.000** | 1 (10.0%) | 14 (14.0%) | 25 (22.7%) |  |
| 550.000 - >700.000*** | 0 (0.0%) | 1 (1.0%) | 1 (0.9%) |  |
| Missing | 2 (20.0%) | 10 (10.0%) | 12 (10.9%) |  |

**Table 4.** Participants’ demographic characteristics split by missing/non-missing alcohol consumption values at six-month follow-up

|  | **Missing**  **(n=10)** | **Non-missing**  **(n=100)** | **Total**  **(N=110)** | ***p*-value** |
| --- | --- | --- | --- | --- |
| **Time since diagnosis** |  |  |  | 0.593 |
| < 6 months | 7 (70.0%) | 55 (55.0%) | 62 (56.4%) |  |
| >12 months | 3 (30.0%) | 45 (45.0%) | 48 (43.6%) |  |
| Missing | 0 | 0 | 0 |  |
| **Etiology** |  |  |  | 0.447 |
| Alcohol | 9 (90.0%) | 92 (92.0%) | 101 (91.8%) |  |
| Methotrexate | 0 | 2 (2.0%) | 2 (1.8%) |  |
| PBC | 0 | 2 (2.0%) | 2 (1.8%) |  |
| AIH | 0 | 2 (2.0%) | 2 (1.8%) |  |
| NASH | 1 (10.0%) | 1 (1.0%) | 2 (1.8%) |  |
| Missing | 0 (0.0%) | 1 (1.0%) | 1 (0.9%) |  |
| **Number of comorbidities** |  |  |  | 0.851 |
| 0-1 | 3 (30.0%) | 44 (44.0%) | 47 (42.7%) |  |
| 2-3 | 5 (50.0%) | 44 (44.0%) | 49 (44.5%) |  |
| >4 | 2 (20.0%) | 12 (12.0%) | 14 (12.7%) |  |
| Missing | 0 | 0 | 0 |  |
| **Child-Pugh** |  |  |  | 0.205 |
| B(8-9) | 3 (30.0%) | 50 (50.0%) | 53 (48.2%) |  |
| C(>9) | 7 (70.0%) | 48 (48.0%) | 55 (50.0%) |  |
| Missing | 0 | 2 (2.0%) | 2 (1.8%) |  |
| **Alcohol consumption at inclusion** |  |  |  | 0.568 |
| No | 4 (40.0%) | 48 (48.0%) | 52 (47.3%) |  |
| Yes | 6 (60.0%) | 49 (49.0%) | 55 (50.0%) |  |
| Missing | 0 | 3 (3.0%) | 3 (2.7%) |  |
| **Reason for admission** |  |  |  | 0.041 |
| Ascites | 8 (80.0%) | 45 (45.0%) | 53 (48.2%) |  |
| HE | 0 | 17 (17.0%) | 17 (15.4%) |  |
| Variceal bleeding | 1 (10.0%) | 15 (15.0%) | 16 (14.5%) |  |
| Electrolyte disturbance | 0 | 12 (12.0%) | 12 (10.9%) |  |
| Icterus | 1 (10.0%) | 8 (8.0%) | 9 (8.2%) |  |
| Bacterial peritonitis | 0 | 2 (2.0%) | 2 (1.8%) |  |
| Missing | 0 | 1 (10.0%) | 1 (0.9%) |  |

**Table 5.** Participants’ demographic characteristics split by missing/non-missing weekly alcohol consumption values at six-month follow-up

|  | **Missing**  **(N=88)** | **Non-missing**  **(N=22)** | **Total**  **(N=110)** | ***p*-value** |
| --- | --- | --- | --- | --- |
| **Age (years)** |  |  |  | 0.085 |
| 36-45 | 2 (2.3%) | 3 (13.6%) | 5 (4.5%) |  |
| 46-55 | 20 (22.7%) | 7 (31.8%) | 27 (24.5%) |  |
| 56-65 | 28 (31.8%) | 7 (31.8%) | 35 (31.8%) |  |
| 66-75 | 31 (35.2%) | 5 (22.7%) | 36 (32.7%) |  |
| 76-85 | 7 (8.0%) | 0 | 7 (6.4%) |  |
| Missing | 0 | 0 | 0 |  |
| **Gender** |  |  |  | 0.445 |
| Female | 44 (50.0%) | 13 (59.1%) | 57 (51.8%) |  |
| Male | 44 (50.0%) | 9 (40.9%) | 53 (48.2%) |  |
| Missing | 0 | 0 | 0 |  |
| **Marital status** |  |  |  | 0.945 |
| Civil partnership | 10 (11.4%) | 4 (18.2%) | 14 (12.7%) |  |
| Divorced | 26 (29.5%) | 6 (27.3%) | 32 (29.1%) |  |
| Married | 26 (29.5%) | 6 (27.3%) | 32 (29.1%) |  |
| Unmarried | 18 (20.5%) | 4 (18.2%) | 22 (20.0%) |  |
| Widowed | 8 (9.1%) | 2 (9.1%) | 10 (9.1%) |  |
| Missing | 0 | 0 | 0 |  |
| **Educational level** |  |  |  | 0.203 |
| Primary school | 19 (21.6%) | 7 (31.8%) | 26 (23.6%) |  |
| Special worker education | 2 (2.3%) | 0 | 2 (1.8%) |  |
| Apprenticeship training | 28 (31.8%) | 7 (31.8%) | 35 (31.8%) |  |
| Shorter theoretical education (1-3 years) | 18 (20.5%) | 4 (18.2%) | 22 (20.0%) |  |
| Longer theoretical education (>3 years) | 13 (14.8%) | 2 (9.1%) | 15 (13.6%) |  |
| Academic education | 7 (7.9%) | 2 (9.1%) | 9 (8.2%) |  |
| Missing | 1 (1.1%) | 0 | 1 (0.9%) |  |
| **Yearly income DKK** |  |  |  | 0.230 |
| 100.000 - 250.000* | 54 (61.4%) | 16 (72.7%) | 70 (63.6%) |  |
| 250.000 - 550.000** | 22 (25.0%) | 3 (13.6%) | 25 (22.7%) |  |
| 550.000 - ­ >700.000*** | 1 (1.1%) | 2 (9.1%) | 3 (2.7%) |  |
| Missing | 11 (12.5%) | 1 (4.6%) | 12 (10.9%) |  |

**Table 6.** Participants’ medical characteristics split by missing/non-missing weekly alcohol consumption values at six-month follow-up

|  | **Missing**  **(N=88)** | **Non-missing**  **(N=22)** | **Total**  **(N=110)** | ***p*-value** |
| --- | --- | --- | --- | --- |
| **Time since diagnosis** |  |  |  | 0.582 |
| < 6 months | 49 (55.7%) | 13 (59.1%) | 62 (56.4%) |  |
| 6-12 months | 39 (44.3%) | 9 (40.9%) | 48 (43.6%) |  |
| Missing | 0 | 0 | 0 |  |
| **Etiology** |  |  |  | 0.784 |
| Alcohol | 79 (89.8%) | 22 (100.0%) | 101 (91.8%) |  |
| Methotrexate | 2 (2.3%) | 0 (0.0%) | 2 (1.8%) |  |
| PBC | 2 (2.3%) | 0 (0.0%) | 2 (1.8%) |  |
| AIH | 2 (2.3%) | 0 (0.0%) | 2 (1.8%) |  |
| NASH | 2 (2.3%) | 0 (0.0%) | 2 (1.8%) |  |
| Missing | 1 (1.1%) | 0 (0.0%) | 1 (0.9%) |  |
| **Number of comorbidities** |  |  |  | 0.357 |
| 0-1 | 38 (43.2%) | 9 (40.9%) | 47 (42.8%) |  |
| 2-3 | 41 (46.6%) | 8 (36.4%) | 49 (44.5%) |  |
| >4 | 9 (10.2%) | 5 (22.7%) | 14 (12.7%) |  |
| Missing | 0 | 0 | 0 |  |
| **Child-Pugh** |  |  |  | 0.006 |
| B(8-9) | 37 (42.0%) | 16 (72.7%) | 53 (48.2%) |  |
| C(>9) | 50 (56.8%) | 5 (22.7%) | 55 (50.0%) |  |
| Missing | 1 (1.1%) | 1 (4.6%) | 2 (1.8%) |  |
| **Alcohol consumption at inclusion** |  |  |  | < 0.001 |
| No | 50 (56.8%) | 2 (9.1%) | 52 (47.3%) |  |
| Yes | 35 (39.8%) | 20 (90.9%) | 55 (50.0%) |  |
| Missing | 3 (3.4) | 0 | 3 (2.7%) |  |
| **Reason for admission** |  |  |  | 0.629 |
| Ascites | 42 (47.7%) | 10 (45.5%) | 52 (47.3%) |  |
| HE | 15 (17.0%) | 2 (9.1%) | 17 (15.5%) |  |
| Variceal bleeding | 13 (14.8%) | 3 (13.6%) | 16 (14.5%) |  |
| Electrolyte disturbance | 7 (6.4%) | 5 (22.7%) | 12 (10.9%) |  |
| Icterus | 7 (6.4%) | 2 (9.1%) | 9 (8.2%) |  |
| Bacterial peritonitis | 2 (1.8%) | 0 | 2 (1.8%) |  |
| Anemia | 1 (0.9%) | 0 | 1 (0.9%) |  |
| Missing | 1 (0.9%) | 0 | 1 (0.9%) |  |
