# Supplementary figures and images for: A randomized controlled trial of a postdischarge nursing intervention for patients with decompensated cirrhosis
Source: Hepatol Commun. 2024 Apr 26;8(5):e0418. doi: 10.1097/HC9.0000000000000418 (PMC12333763; doi:10.1097/HC9.0000000000000418)

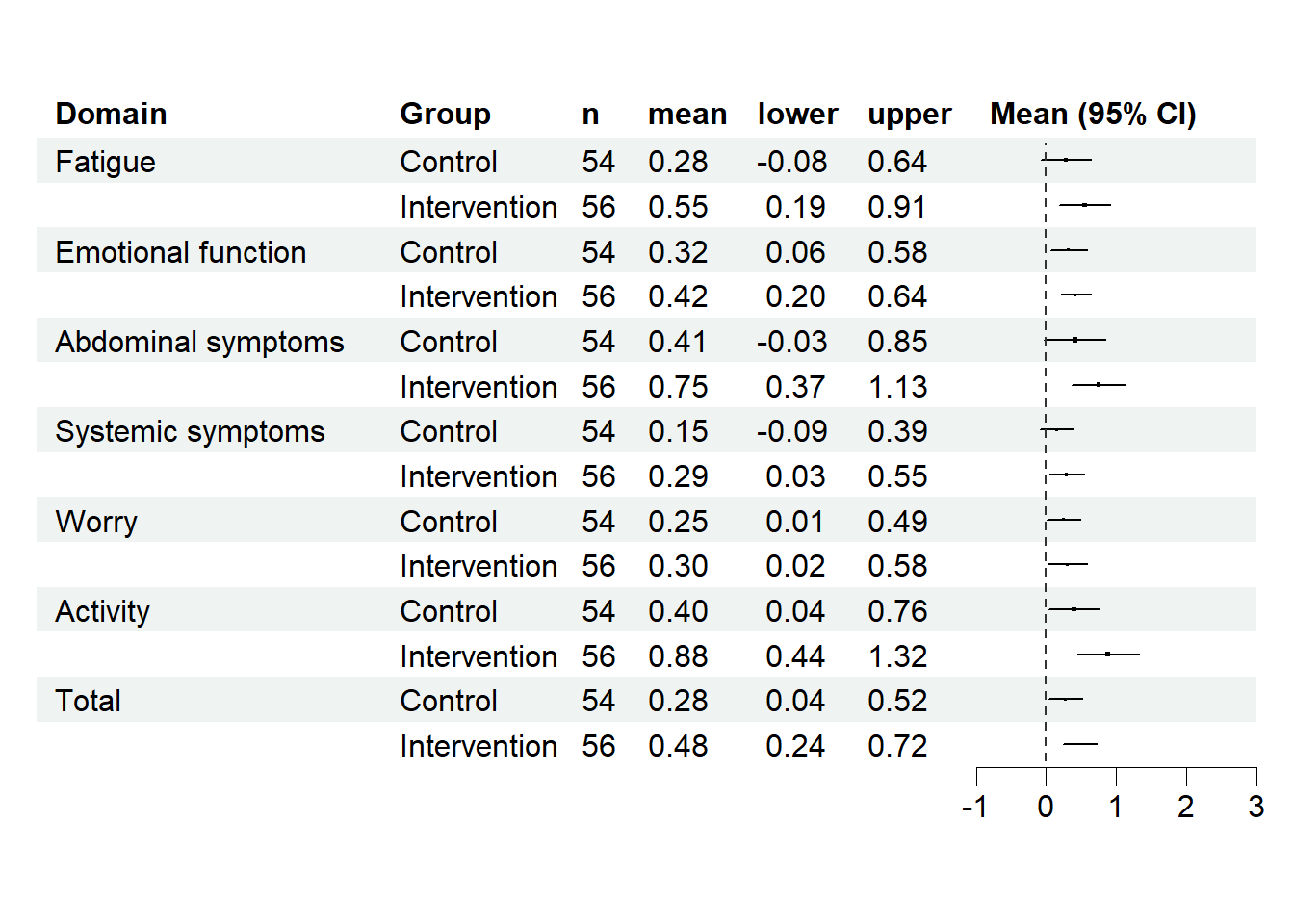

Supplement: SUPPLEMENTARY MATERIAL [file hc9-8-e0418-s004.tiff]
