# Supplementary material for: A randomized controlled trial of a postdischarge nursing intervention for patients with decompensated cirrhosis
Source: Hepatol Commun. 2024 Apr 26;8(5):e0418. doi: 10.1097/HC9.0000000000000418 (PMC12333763; doi:10.1097/HC9.0000000000000418)
Supplement: SUPPLEMENTARY MATERIAL [file hc9-8-e0418-s005.docx]

**SDC 5, Table 7.** Mortality and causes of mortality at six- and 12-months follow-up.

|  | As-treated analysis | | | | Intention-to-treat analysis | | | |
| --- | --- | --- | --- | --- | --- | --- | --- | --- |
|  | Control (N=44) | Intervention (N=41) | Total (N=85) | *P*-value | Control (N=54) | Intervention (N=56) | Total (N=110) | *P*-value |
| Number of patients dead at six-months follow-up | 9 | 9 | 18 | 0.912 | 14 | 21 | 35 | 0.193 |
| Number of patients dead at 12-months follow-up | 14 | 12 | 26 | 0.931 | 19 | 25 | 44 | 0.311 |
| Causes of death at 12-months follow-up  Hepatic failure  Hepatorenal syndrome  Infection  Variceal bleeding  Traumatic injury/fall  Cancer  Unknown reasons | 4  5  2  2  -  -  1 | 5  2  3  -  1  -  1 | 9  7  5  2  1  -  2 | -  -  -  -  -  -  - | -  6  3  3  -  2  - | 8  5  4  1  2  4  - | 8  11  7  4  2  6  - | -  -  -  -  -  -  - |
