# Supplementary material for: A randomized controlled trial of a postdischarge nursing intervention for patients with decompensated cirrhosis
Source: Hepatol Commun. 2024 Apr 26;8(5):e0418. doi: 10.1097/HC9.0000000000000418 (PMC12333763; doi:10.1097/HC9.0000000000000418)
Supplement: SUPPLEMENTARY MATERIAL [file hc9-8-e0418-s006.docx]

**SDC 6, Table 8.** Changes in alcohol consumption at six-month follow-up.

|  | As-treated analysis | | | | Intention-to-treat analysis | | | |
| --- | --- | --- | --- | --- | --- | --- | --- | --- |
|  | Control (n=44) | Intervention (n=41) | Total (N=85) | *P-value* | Control (n=54) | Intervention (n=56) | Total (N=110) | *P-value* |
| Alcohol consumption baseline |  |  |  | 0.541 |  |  |  | 0.241 |
| No | 12 (27.3%) | 13 (31.7%) | 25 (29.4%) |  | 14 (25.9%) | 21 (37.5%) | 35 (31.8%) |  |
| Yes | 32 (72.7%) | 28 (68.3%) | 60 (70.6%) |  | 40 (74.1%) | 35 (62.5%) | 75 (68.2%) |  |
| Missing | 0 | 0 | 0 |  | 0 | 0 | 0 |  |
| Alcohol consumption EOS |  |  |  | **< 0.001** |  |  |  | **< 0.001** |
| No | 16 (36.4%) | 30 (73.2%) | 46 (54.1%) |  | 18 (33.3%) | 39 (69.6%) | 57 (51.8%) |  |
| Yes | 28 (63.6%) | 11 (26.8%) | 39 (45.9%) |  | 36 (66.7%) | 17 (30.4%) | 53 (48.2%) |  |
| Missing | 0 | 0 | 0 |  | 0 | 0 | 0 |  |
| Weekly alcohol consumption baseline |  |  |  | 0.412 |  |  |  | 0.213 |
| Alcohol dependence | 16 (36.4%) | 10 (24.4%) | 26 (30.6%) |  | 22 (40.7%) | 17 (30.4%) | 39 (35.5%) |  |
| Heavy drinking (>7 units of alcohol weekly for women and >14 for men) | 10 (22.7%) | 7 (17.1%) | 17 (20.0%) |  | 11 (20.3%) | 7 (12.5%) | 18 (16.4%) |  |
| Moderate drinking (<7 units of alcohol weekly for women and <14 for men) | 3 (6.8%) | 6 (14.6%) | 9 (10.6%) |  | 3 (5.6%) | 7 (12.5%) | 10 (9.1%) |  |
| Abstinent | 12 (27.3%) | 13 (31.7) | 25 (29.4%) |  | 15 (27.8%) | 20 (35.7%) | 35 (31.8%) |  |
| Missing | 3 (6.8%) | 5 (12.2%) | 8 (9.4%) |  | 3 (5.6%) | 5 (8.9%) | 8 (7.2%) |  |
| Weekly alcohol consumption EOS |  |  |  | 0.488 |  |  |  | 0.303 |
| Alcohol dependence | 13 (29.5%) | 9 (22.0%) | 22 (25.9%) |  | 19 (35.2%) | 16 (28.6%) | 35 (31.8%) |  |
| Heavy drinking (>7 units of alcohol weekly for women and >14 for men) | 9 (20.5%) | 8 (19.5%) | 17 (20.0%) |  | 10 (18.5%) | 8 (14.3%) | 18 (16.4%) |  |
| Moderate drinking (<7 units of alcohol weekly for women and <14 for men) | 7 (15.9%) | 6 (14.6%) | 13 (15.3%) |  | 7 (12.9%) | 7 (12.5%) | 14 (12.7%) |  |
| Abstinent | 12 (27.3%) | 18 (43.9%) | 30 (35.3%) |  | 15 (27.8%) | 25 (44.6%) | 40 (36.4%) |  |
| Missing | 3 (6.8%) | 0 | 3 (3.5%) |  | 3 (5.6) | 0 | 3 (2.7%) |  |
| Change in baseline alcohol consumption |  |  |  | **0.003** |  |  |  | **0.002** |
| Continued drinking (Yes -> Yes) | 26 (59.1%) | 11 (26.8%) | 37 (43.5%) |  | 33 (61.1%) | 18 (32.1%) | 51 (46.4%) |  |
| Stopped drinking (Yes -> No) | 6 (13.6%) | 17 (41.5%) | 23 (27.1%) |  | 6 (11.1%) | 18 (32.1%) | 24 (21.8%) |  |
| Broke abstinence (No -> Yes) | 2 (4.5%) | 0 | 2 (2.4%) |  | 2 (3.7%) | 0 | 2 (1.8%) |  |
| Remained abstinent (No -> No) | 10 (22.7%) | 14 (31.7%) | 24 (27.1%) |  | 13 (24.1) | 20 (35.8) | 33 (30.0%) |  |
| Missing | 0 | 0 | 0 |  | 0 | 0 | 0 |  |
